# Supplementary material for: Blackberry-Loaded AgNPs Attenuate Hepatic Ischemia/Reperfusion Injury via PI3K/Akt/mTOR Pathway
Source: Metabolites. 2023 Mar 13;13(3):419. doi: 10.3390/metabo13030419 (PMC10051224; doi:10.3390/metabo13030419)
Supplement: Supplementary file 1 [file metabolites-13-00419-s001.zip › metabolites-2252411-supplementary.pdf]

Supplementary Material
Blackberry-loaded AgNPs Attenuate Hepatic Ischemia/Reperfusion Injury via PI3K/Akt/mTOR Pathway

Ahmed M. Fathi[a], Shaimaa Waz[a], Eman Alaaeldin[b, c], Nisreen D. M. Toni[d], Ahmed M. Sayed[e] Usama Ramadan Abdelmohsen[f, g]*, Maiiada H. Nazmy[a]*

[a] Department of Biochemistry, Faculty of Pharmacy, Minia University, Minia 61519, Egypt

[b] Department of Pharmaceutics, Faculty of Pharmacy, Minia University, Minia 61519, Egypt,

[c] Department of Pharmaceutics, Faculty of Pharmacy, Deraya University, Minia, 61111, Egypt,

[d] Department of Pathology, Faculty of Medicine, Minia University, Minia 61519, Egypt

[e] Department of Pharmacognosy, Faculty of Pharmacy, Nahda University, 62513 Beni-Suef, Egypt.

[f] Department of Pharmacognosy, Faculty of Pharmacy, Minia University, Minia 61519, Egypt

[g] Department of Pharmacognosy, Faculty of Pharmacy, Deraya University, New Minia 61111, Egypt,

Correspondence: mnazmy@yahoo.com and Usama.ramadan@mu.edu.eg

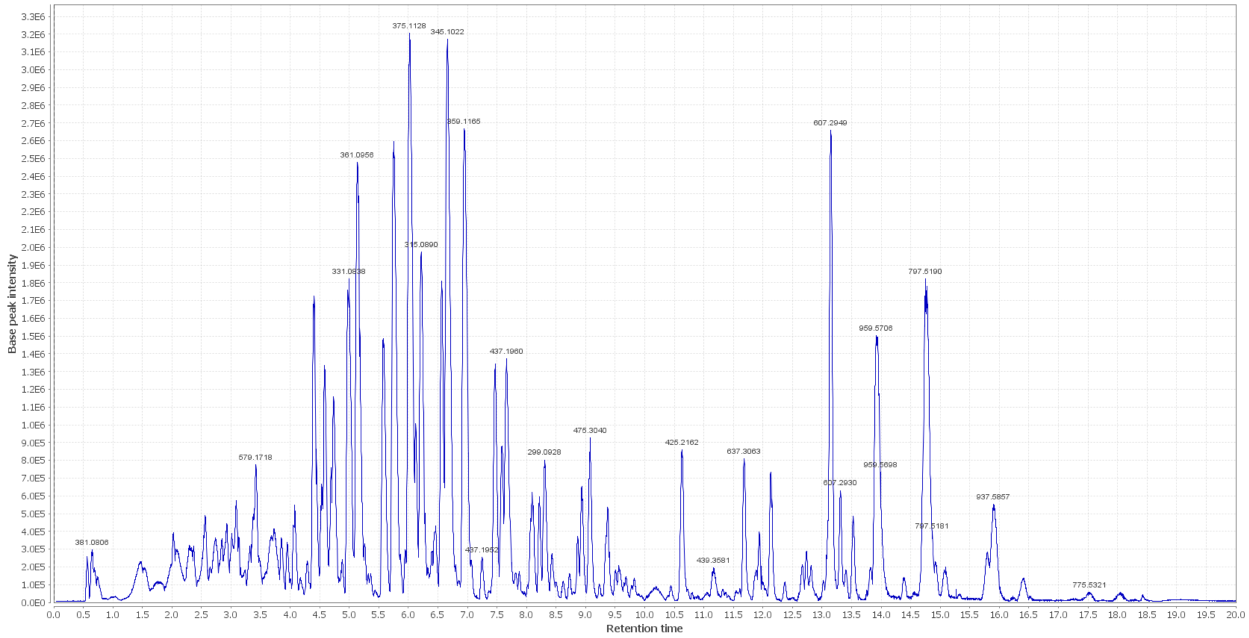

Figure S1. Total ion chromatogram of crude extract of Blackberry leaves (Positive mode)

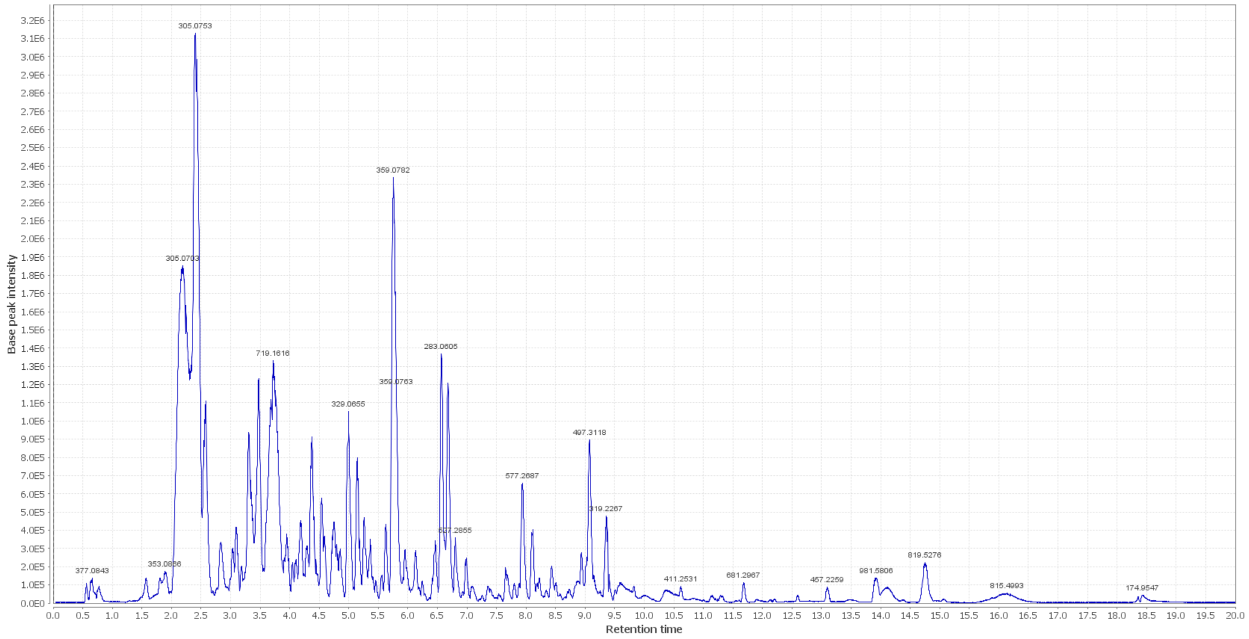

Figure S2. Total ion chromatogram of crude extract of Blackberry leaves (Negative mode)

ALT RESULTS

	Standard 1	Standard 2	Standard 3	Standard 4	Standard 5		
**Conc**	0	25	50	83	126		
**Absorpance**	0.536	0.681	0.79	0.896	1.009		
**A-BLANK**	0	0.145	0.254	0.36	0.473		
	**Absorbance**						
	**Sham**	**IRI**	**BBE**	**AgNPs**	**200 BBE-AgNPs**	**50 BBE-AgNPs**	**Silymarin**
**Rat 1**	0.668	1.024	0.958	1.017	0.808	0.666	0.751
**Rat 2**	0.676	1.018	0.946	1.002	0.865	0.805	0.724
**Rat 3**	0.644	1.098	0.916	0.919	0.794	0.804	0.837
**Rat 4**	0.659	1.036	0.932	1.129	0.884	0.695	0.783
**Rat 5**	0.656	1.024	0.823	0.933	0.828	0.711	0.823
**Rat 6**	0.671	1.017	0.931	1.053	0.702	0.698	0.876
	**Serum ALT Level**						
**Conc 1**	321.9512	1190.244	1029.26829	1173.171	663.414634	317.07317	524.3902
**Conc 2**	341.4634	1175.61	1000	1136.585	802.439024	656.09756	458.5366
**Conc 3**	263.4146	1370.732	926.829268	934.1463	629.268293	653.65854	734.1463
**Conc 4**	300	1219.512	965.853659	1446.341	848.780488	387.80488	602.439
**Conc 5**	292.6829	1190.244	700	968.2927	712.195122	426.82927	700
**Conc 6**	329.2683	1173.171	963.414634	1260.976	404.878049	395.12195	829.2683
**Mean**	308.1301	1219.919	930.894309	1153.252	676.829268	472.76423	641.4634
**Std. Deviation**	28.47154	75.7028	118.373537	189.9461	156.840837	145.55412	138.5486
**SEM**	11.62346	30.90554	48.3257941	77.54517	64.0300036	59.422219	56.56223

Table S1. Effect of pretreatment with BBE, AgNPs, 200 or 50 BBE-AgNPs and silymarin on serum ALT level in hepatic I/R injured rats. Values are presented as means of 6 animals ± SEM. Data were analyzed using one-way ANOVA followed by Tukey's Multiple Comparisons. ALT, alanine transaminase; BBE, Blackberry Extract; IRI, ischemia/reperfusion injury; AgNPs, Silver Nanoparticles; BBE-AgNPs, Blackberry loaded Silver Nanoparticles. Absorbance of samples measured by spectrophotometer at γ = 505 nm and concentration of serum ALT of each sample was calculated according to the kit.

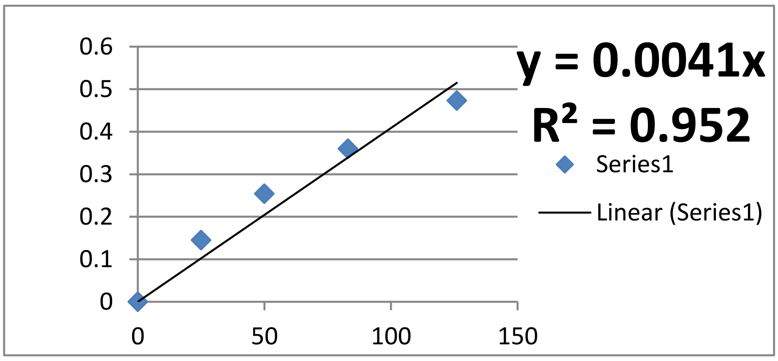

AST RESULTS

Conc	0	22	55	95	150		
Absorpance	0.553	0.64	0.813	0.924	1.055		
A-BLANK	0	0.087	0.26	0.371	0.502		
	Absorbance						
	Normal	IRI	BBE	Empty	200 AgNPs	50 AgNPs	Silymarin
Rat 1	0.721	1.156	1.067	1.029	0.964	0.787	0.921
Rat 2	0.684	1.166	1.134	1.074	1.005	0.867	0.852
Rat 3	0.681	1.234	1.154	0.996	0.899	0.833	0.924
Rat 4	0.696	1.236	1.068	1.022	0.998	0.815	0.839
Rat 5	0.711	1.177	1.168	1.114	0.934	0.812	0.942
Rat 6	0.632	1.184	1.114	1.043	0.945	0.862	0.911
	Serum ALT Level						
Conc 1	466.6667	1675	1427.77778	1322.222	1141.66667	650	1022.222
Conc 2	363.8889	1702.778	1613.88889	1447.222	1255.55556	872.22222	830.5556
Conc 3	355.5556	1891.667	1669.44444	1230.556	961.111111	777.77778	1030.556
Conc 4	397.2222	1897.222	1430.55556	1302.778	1236.11111	727.77778	794.4444
Conc 5	438.8889	1733.333	1708.33333	1558.333	1058.33333	719.44444	1080.556
Conc 6	219.4444	1752.778	1558.33333	1361.111	1088.88889	858.33333	994.4444
Mean	373.6111	1775.463	1568.05556	1370.37	1123.61111	767.59259	958.7963
Std. Deviation	86.79389	95.91046	118.942745	116.4107	111.592706	86.042386	117.2396
SEM	35.43346	39.15528	48.5581722	47.52445	45.5575316	35.126657	47.86285

Table S2. Effect of pretreatment with BBE, AgNPs, 200 or 50 BBE-AgNPs and silymarin on serum AST level in hepatic I/R injured rats. Values are presented as means of 6 animals ± SEM. Data were analyzed using one-way ANOVA followed by Tukey's Multiple Comparisons. AST, aspartate transaminase; BBE, Blackberry Extract; IRI, ischemia/reperfusion injury; AgNPs, Silver Nanoparticles; BBE-AgNPs, Blackberry loaded Silver Nanoparticles. Absorbance of samples measured by spectrophotometer at γ = 505 nm and concentration of serum AST of each sample was calculated according to the kit.

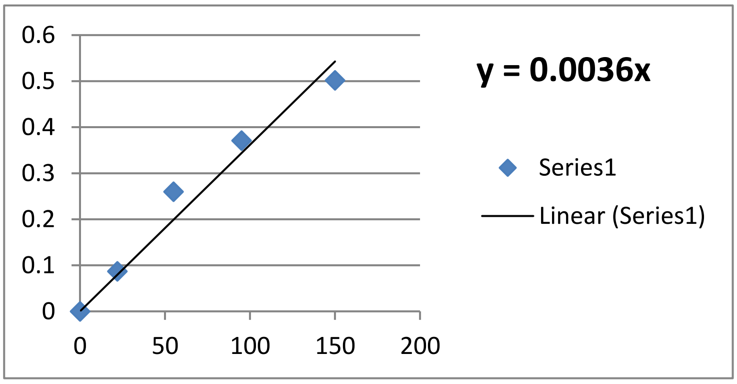

MDA RESULTS

		Standard	0.295	Sample Blank		0.14	
		St Blank	100				
	**Absorbance**						
	**Normal**	**IRI**	**BBE**	**Empty**	**200 AgNPs**	**50 AgNPs**	**Silymarin**
**Rat 1**	0.505	0.741	0.653	0.717	0.581	0.479	0.581
**Rat 2**	0.465	0.799	0.646	0.688	0.631	0.519	0.534
**Rat 3**	0.478	0.722	0.583	0.694	0.585	0.525	0.488
**Rat 4**	0.527	0.691	0.573	0.678	0.624	0.485	0.464
**Rat 5**	0.434	0.739	0.638	0.712	0.536	0.508	0.471
**Rat 6**	0.472	0.731	0.564	0.652	0.611	0.537	0.468
	**Liver MDA Level**						
**Conc 1**	309.322	509.322	434.7458	488.9831	373.7288	287.2881	373.7288
**Conc 2**	275.4237	558.4746	428.8136	464.4068	416.1017	321.1864	333.8983
**Conc 3**	286.4407	493.2203	375.4237	469.4915	377.1186	326.2712	294.9153
**Conc 4**	327.9661	466.9492	366.9492	455.9322	410.1695	292.3729	274.5763
**Conc 5**	249.1525	507.6271	422.0339	484.7458	335.5932	311.8644	280.5085
**Conc 6**	281.3559	500.8475	359.322	433.8983	399.1525	336.4407	277.9661
**Mean**	286.1985	514.5278	399.7579	457.9903	386.3196	311.3801	304.3584
**SEM**	9.693802	13.34889	11.9473	10.78615	10.32042	6.794418	13.81472
**Std. Deviation**	25.64739	35.31784	31.60958	28.53747	27.30527	17.97634	36.55032

Table S3. Effect of pretreatment with BBE, AgNPs, 200 or 50 BBE-AgNPs and silymarin on tissue MDA level in hepatic I/R injured rats. Values are presented as means of 6 animals ± SEM. Data were analyzed using one-way ANOVA followed by Tukey's Multiple Comparisons. MDA, malondialdehyde ; BBE, Blackberry Extract; IRI, ischemia/reperfusion injury; AgNPs, Silver Nanoparticles; BBE-AgNPs, Blackberry loaded Silver Nanoparticles. Absorbance of samples measured by spectrophotometer at γ = 534 nm and concentration of tissue MDA of each sample was calculated according to the kit.

GSH RESULTS

	Absorbance						
	Normal	IRI	BBE	Empty	200 AgNPs	50 AgNPs	Silymarin
Rat 1	0.18	0.112	0.122	0.132	0.132	0.158	0.167
Rat 2	0.174	0.115	0.131	0.124	0.151	0.165	0.165
Rat 3	0.172	0.122	0.123	0.121	0.143	0.159	0.169
Rat 4	0.169	0.111	0.13	0.123	0.134	0.165	0.165
Rat 5	0.173	0.119	0.136	0.122	0.141	0.16	0.175
Rat 6	0.179	0.109	0.125	0.115	0.152	0.165	0.167
	Liver GSH Level						
Conc 1	52.614	7.326	13.986	20.646	20.646	37.962	43.956
Conc 2	48.618	9.324	19.98	15.318	33.3	42.624	42.624
Conc 3	47.286	13.986	14.652	13.32	27.972	38.628	45.288
Conc 4	45.288	6.66	19.314	14.652	21.978	42.624	42.624
Conc 5	47.952	11.988	23.31	13.986	26.64	39.294	49.284
Conc 6	51.948	5.328	15.984	9.324	33.966	42.624	43.956
Mean	48.80829	9.609429	18.55286	15.12771	27.68657	40.626	44.71714
Std. deviation	2.599798	3.326827	3.756236	3.68241	5.113578	2.034664	2.288708
SEM	0.982631	1.257422	1.419724	1.39182	1.932751	0.769031	0.86505

Table S4. Effect of pretreatment with BBE, AgNPs, 200 or 50 BBE-AgNPs and silymarin on tissue GSH level in hepatic I/R injured rats. Values are presented as means of 6 animals ± SEM. Data were analyzed using one-way ANOVA followed by Tukey's Multiple Comparisons. GSH, reduced glutathione ; BBE, Blackberry Extract; IRI, ischemia/reperfusion injury; AgNPs, Silver Nanoparticles; BBE-AgNPs, Blackberry loaded Silver Nanoparticles. Absorbance of samples measured by spectrophotometer at γ = 405 nm and concentration of tissue GSH of each sample was calculated according to the kit.

SOD RESULTS

	Absorbance						
	Normal	IRI	BBE	Empty	200 AgNPs	50 AgNPs	Silymarin
Rat 1	28	73	37	68	45	34	28
Rat 2	30	82	64	58	53	38	29
Rat 3	22	79	63	60	50	25	27
Rat 4	38	74	54	62	62	37	33
Rat 5	33	87	48	76	54	40	39
Rat 6	29	73	64	66	60	30	30
		% Inhibition					
Conc 1	77.6	41.6	70.4	45.6	64	72.8	77.6
Conc 2	76	34.4	48.8	53.6	57.6	69.6	76.8
Conc 3	82.4	36.8	49.6	52	60	80	78.4
Conc 4	69.6	40.8	56.8	50.4	50.4	70.4	73.6
Conc 5	73.6	30.4	61.6	39.2	56.8	68	68.8
Conc 6	76.8	41.6	48.8	47.2	52	76	76
		Liver SOD Activity					
	Normal	IRI	BBE	Empty	200 AgNPs	50 AgNPs	Silymarin
	291	156	264	171	240	273	291
	285	129	183	201	216	261	288
	309	138	186	195	225	300	294
	261	153	213	189	189	264	276
	276	114	231	147	213	255	258
	288	156	183	177	195	285	285
Mean	285	141	210	180	213	273	282
SEM	6.526868	7.014271	13.41641	8.01249	7.70714	6.884766	5.422177
St Dev	15.9875	17.18139	32.86335	19.62651	18.87856	16.86416	13.28157

Table S5. Effect of pretreatment with BBE, AgNPs, 200 or 50 BBE-AgNPs and silymarin on tissue SOD activity in hepatic I/R injured rats. Values are presented as means of 6 animals ± SEM. Data were analyzed using one-way ANOVA followed by Tukey's Multiple Comparisons. SOD, superoxide dismutase ; BBE, Blackberry Extract; IRI, ischemia/reperfusion injury; AgNPs, Silver Nanoparticles; BBE-AgNPs, Blackberry loaded Silver Nanoparticles. Absorbance of samples measured by spectrophotometer at γ = 560 nm and activity of tissue SOD of each sample was calculated according to the kit.

CAT RESULTS

	Absorbance						
	**Normal**	**IRI**	**BBE**	**Empty**	**200 AgNPs**	**50 AgNPs**	**Silymarin**
**Rat 1**	0.861	0.712	0.765	0.769	0.806	0.825	0.858
**Rat 2**	0.852	0.722	0.736	0.793	0.784	0.831	0.843
**Rat 3**	0.881	0.728	0.777	0.786	0.812	0.811	0.854
**Rat 4**	0.877	0.731	0.734	0.813	0.818	0.829	0.862
**Rat 5**	0.849	0.736	0.764	0.782	0.799	0.823	0.863
**Rat 6**	0.859	0.728	0.762	0.775	0.804	0.812	0.852
	**Liver CAT Activity**						
**Conc 1**	56.09137	18.27411	31.72589	32.74112	42.13198	46.95431	55.32995
**Conc 2**	53.80711	20.81218	24.36548	38.83249	36.54822	48.47716	51.52284
**Conc 3**	61.16751	22.33503	34.77157	37.05584	43.65482	43.40102	54.31472
**Conc 4**	60.15228	23.09645	23.85787	43.90863	45.17766	47.96954	56.34518
**Conc 5**	53.04569	24.36548	31.47208	36.04061	40.35533	46.4467	56.59898
**Conc 6**	55.58376	22.33503	30.96447	34.26396	41.62437	43.65482	53.80711
**Mean**	57.7955	21.79115	28.97027	36.62074	42.05946	45.97534	55.22117
**Std. Deviation**	4.304043	1.935316	4.280818	3.848378	2.998486	2.022131	2.283592
**SEM**	1.626776	0.731481	1.617997	1.45455	1.133321	0.764294	0.863117

Table S6. Effect of pretreatment with BBE, AgNPs, 200 or 50 BBE-AgNPs and silymarin on tissue CAT activity in hepatic I/R injured rats. Values are presented as means of 6 animals ± SEM. Data were analyzed using one-way ANOVA followed by Tukey's Multiple Comparisons. CAT, catalase ; BBE, Blackberry Extract; IRI, ischemia/reperfusion injury; AgNPs, Silver Nanoparticles; BBE-AgNPs, Blackberry loaded Silver Nanoparticles. Absorbance of samples measured by spectrophotometer at γ = 510 nm and activity of tissue CAT of each sample was calculated according to the kit.

WESTERN BLOTTING

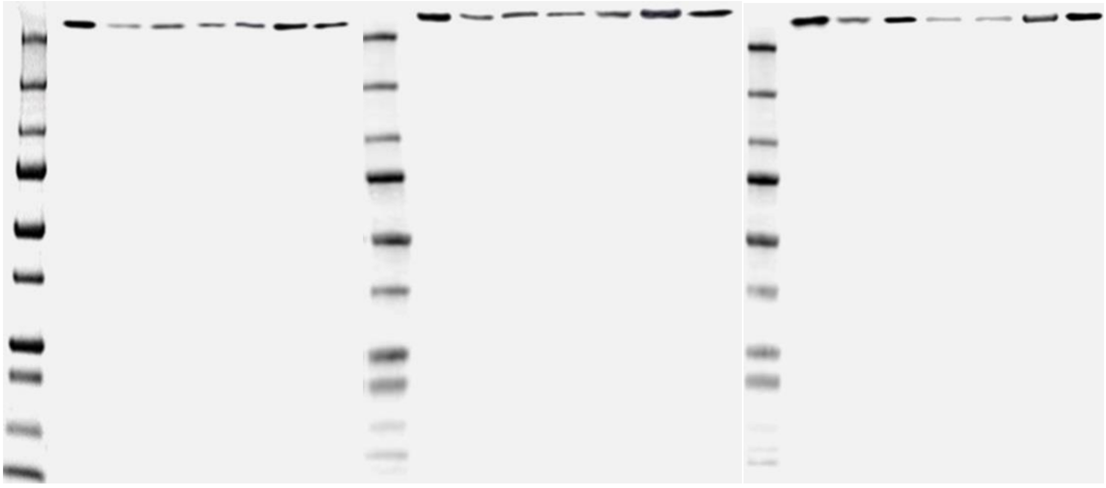

Figure S3. Western blotting of p-mTOR original images

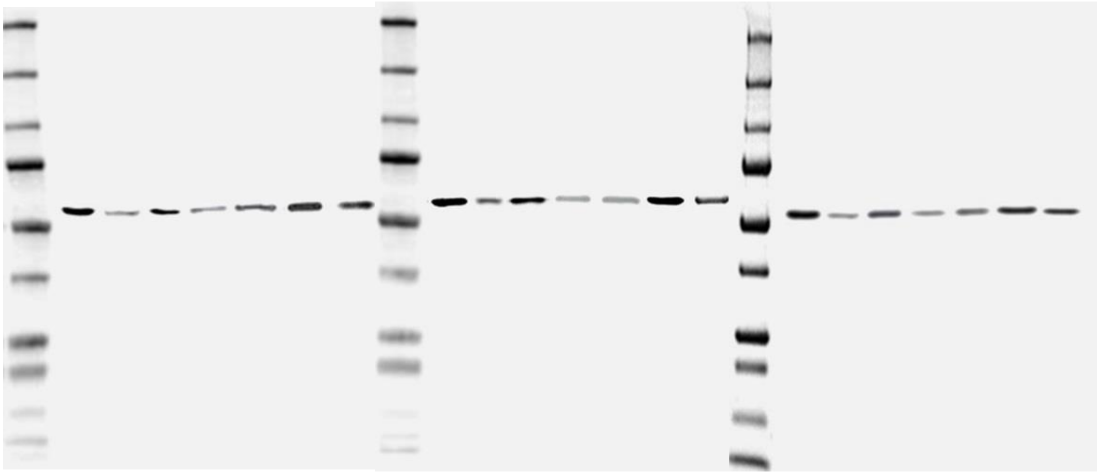

Figure S4. Western blotting of p-Akt original images

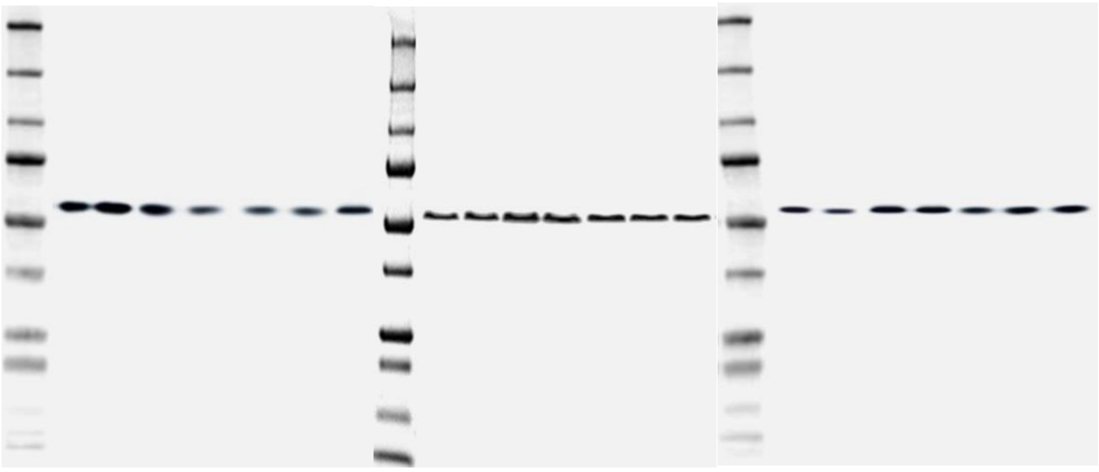

Figure S5. Western blotting Akt original images

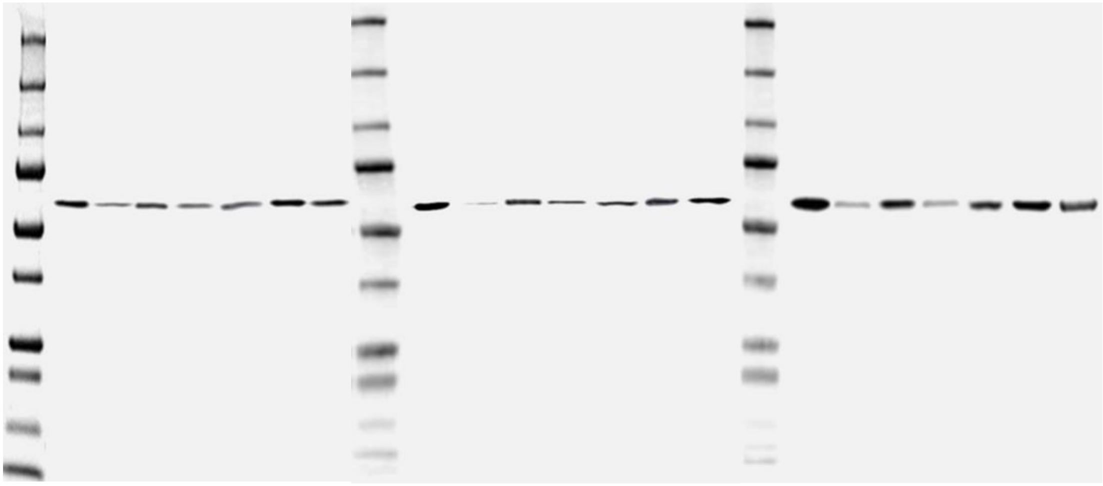

Figure S6. Western blotting of p-PI3k original images

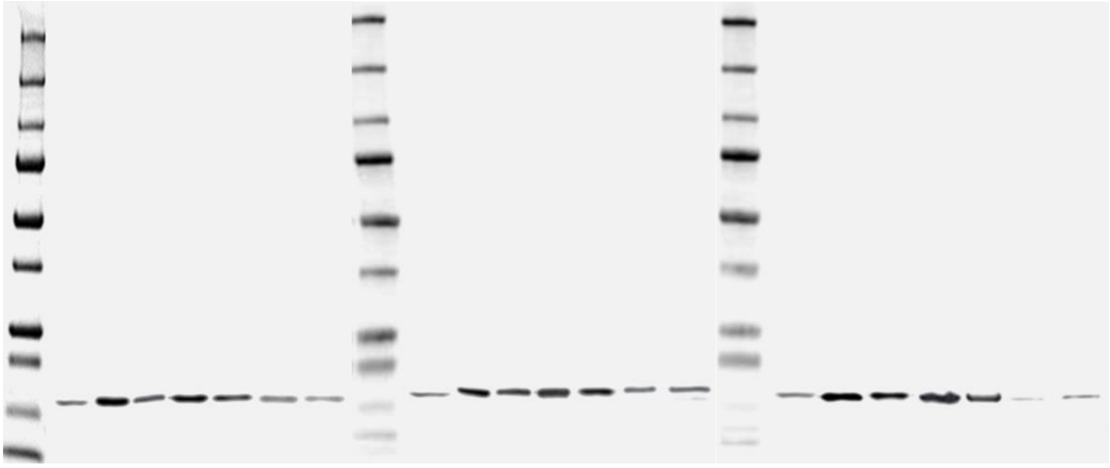

Figure S7. Western blotting of Cleaved Caspase-3 original images

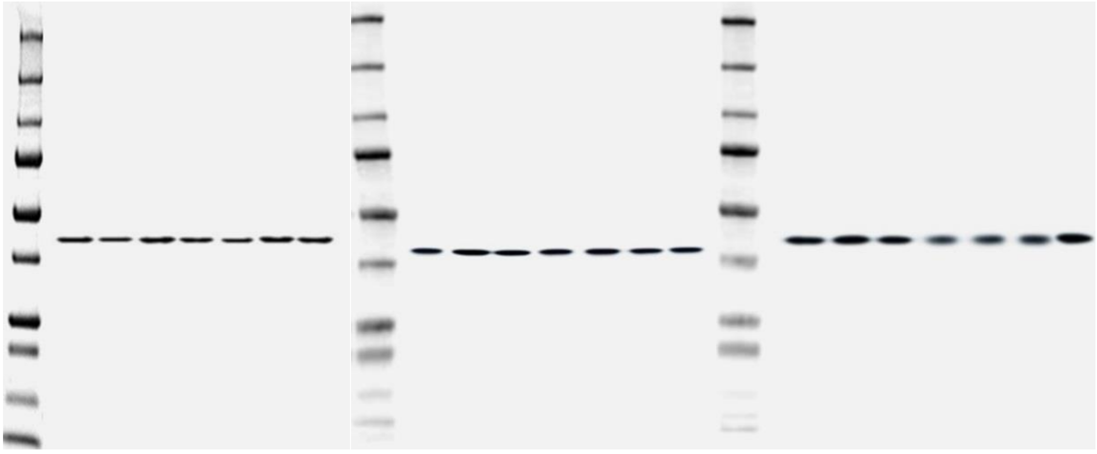

Figure S8. Western blotting of B-Actin original images

IMMUNOHISTOCHEMISTRY

% Area of Positive Bax Staining

	Sham	IRI	BBE	AgNPs	200 BBE-AgNPs	50 BBE-AgNPs	Silymarin
Rat 1	2.1	12.7	6.6	10.3	5.5	2.4	3.4
Rat 2	1.5	12.9	6	11.1	4.2	3.4	2
Rat 3	1.2	12.5	7	12	3.5	4.5	1.5
Rat 4	0.7	12.2	6.1	9.1	3.8	1.8	2.8
Rat 5	1.7	11.5	5.9	10.2	4	3	1.6
Rat 6	1.3	11.1	7.2	13.3	5.3	3.1	2.5
Mean	1.417	12.15	6.467	11.00	4.383	3.033	2.300
SEM	0.1939	0.2895	0.2246	0.6066	0.3361	0.3748	0.3011
Std. Deviation	0.4750	0.7092	0.5502	1.486	0.8232	0.9180	0.7376

Table S7. Effect of pretreatment with BBE, AgNPs, 200 or 50 BBE-AgNPs and silymarin on the expression of Bax in hepatic I/R injured rats. Values are presented as means of 6 animals ± SEM. Data were analyzed using one-way ANOVA followed by Tukey's Multiple Comparisons. Bax, BCL2-Associated X Protein; BBE, Blackberry Extract; IRI, ischemia/reperfusion injury; AgNPs, Silver Nanoparticles; BBE-AgNPs, Blackberry loaded Silver Nanoparticles. **Percentage area of positive Bax staining was measured by the programme Image J.**

% Area of Positive Caspase-9 Staining

	Sham	IRI	BBE	AgNPs	200 BBE-AgNPs	50 BBE-AgNPs	Silymarin
Rat 1	0.4	14.5	7	10.1	6.7	3.2	0.9
Rat 2	1.1	13.4	6.3	11	5	4.5	1
Rat 3	0.2	13	10.2	11.2	4.3	4.7	3.3
Rat 4	1.4	13.2	9.3	13.7	4.7	4.8	2
Rat 5	2.7	12	6.5	11.4	5.2	5.4	4.3
Rat 6	0.5	12.4	5.5	12	5.6	3.6	1.7
Mean	1.050	13.08	7.467	11.57	5.250	4.367	2.200
SEM	0.3784	0.3544	0.7575	0.4958	0.3413	0.3333	0.5489
Std. Deviation	0.9268	0.8681	1.855	1.214	0.8361	0.8165	1.345

Table S8. Effect of pretreatment with BBE, AgNPs, 200 or 50 BBE-AgNPs and silymarin on the expression of Caspase-9 in hepatic I/R injured rats. Values are presented as means of 6 animals ± SEM. Data were analyzed using one-way ANOVA followed by Tukey's Multiple Comparisons. BBE, Blackberry Extract; IRI, ischemia/reperfusion injury; AgNPs, Silver Nanoparticles; BBE-AgNPs, Blackberry loaded Silver Nanoparticles. **Percentage area of positive Caspase-9 staining was measured by the programme Image J.**

Methods

1. Molecular Dynamics Simulation

Desmond v. 2.2 software was used for performing MDS experiments [1-3]. This software applies the OPLS force field. Protein systems were built using the System Builder option, where the protein structure was embedded in an orthorhombic box of TIP3P water together with 0.15 M Na^+ and Cl^- ions in a 20 $Å^3$ solvent buffer. Afterward, the prepared systems were energy minimized and equilibrated for 10 ns. Desmond software automatically parameterizes inputted ligands during the system-building step according to the OPLS force field. Metal-containing proteins like PLA_2 that contain Ca^{2+} ion in the active site should be parameterized during the protein preparation step. To do so, a hetero state should be generated for hetero atoms like Ca (Generate Hetero States). This function is a part of the maestro's Protein Preparation wizard. This step will enable the formation of a suitable hetero state or co-ordinate covalent state for the heteroatom (i.e. Ca^{2+}) in complex with the protein so that force fields like OPLS can easily recognize the zinc atom.

For simulations performed by NAMD [4], the parameters and topologies of the compounds were calculated either using the Charmm27 force field with the online software Ligand Reader and Modeler (http://www.charmm-gui. org/?doc=input/ligandrm, accessed on 4 September 2022) [5] or using the VMD plugin Force Field Toolkit (ffTK). Afterward, the generated parameters and topology files were loaded to VMD to readily read the protein-ligand complexes without errors and then conduct the simulation step. Harmonic Tcl forces were applied to keep Ca^{2+} in place.

2. Binding Free Energy Calculations

Binding free energy calculations ($\Delta G_{\text{binding}}$) were performed using the free energy perturbation (FEP) method [4]. This method was described in detail in a recent article by Kim and coworkers [4]. Briefly, this method calculates the binding free energy $\Delta G_{\text{binding}}$ according to the following equation: $\Delta G_{\text{binding}} = \Delta G_{\text{Complex}} - \Delta G_{\text{Ligand}}$. The value of each ΔG is estimated from a separate simulation using NAMD software. Interestingly, all input files required for simulation by NAMD can be papered by using the online website CharmmGUI (https://charmm-gui.org/?doc=input/afes.abinding, accessed on 18 September 2022). Subsequently, we can use these files in NAMD to produce the required simulations using the FEP calculation function in NAMD. The equilibration was achieved in the NPT ensemble at 310 K and 1 atm (1.01325 bar) with Langevin piston pressure (for “Complex” and “Ligand”) in the presence of the TIP3P water model. Then, 10 ns FEP simulations were performed for each compound, and the last 5 ns of the free energy values were measured for the final free energy values [4]. Finally, the generated trajectories were visualized and analyzed using VMD software [5].

References

1. Bowers, K.J., et al. *Scalable algorithms for molecular dynamics simulations on commodity clusters*. in *Proceedings of the 2006 ACM/IEEE Conference on Supercomputing*. 2006.
2. Release, S., *3: Desmond molecular dynamics system, DE Shaw research, New York, NY, 2017.* Maestro-Desmond Interoperability Tools, Schrödinger, New York, NY, 2017.
3. Phillips, J.C., et al., *Scalable molecular dynamics with NAMD.* Journal of computational chemistry, 2005. **26**(16): p. 1781-1802.
4. Kim, S., et al., *CHARMM-GUI free energy calculator for absolute and relative ligand solvation and binding free energy simulations.* Journal of chemical theory and computation, 2020. **16**(11): p. 7207-7218.
5. Ngo, S.T., et al., *Benchmark of popular free energy approaches revealing the inhibitors binding to SARS-CoV-2 Mpro.* Journal of chemical information and modeling, 2021. **61**(5): p. 2302-2312.
